# Direct Enzymatic Glucose/O_2_ Biofuel Cell based on Poly-Thiophene Carboxylic Acid alongside Gold Nanostructures Substrates Derived through Bipolar Electrochemistry

**DOI:** 10.1038/s41598-018-32893-2

**Published:** 2018-10-10

**Authors:** Fereshte Gholami, Aso Navaee, Abdollah Salimi, Rezgar Ahmadi, Azam Korani, Rahman Hallaj

**Affiliations:** 1 Department of Chemistry, University of Kurdistan, 66177-15175 Sanandaj, Iran; 2 Research Centre for Nanotechnology, University of Kurdistan, 66177-15175 Sanandaj, Iran; 3 Vice chancellor for Food and Drug, Kurdistan University of Medical Sciences, Sanandaj, Iran

## Abstract

Bipolar electrochemistry (BPE) has been lately explored as a simple, reliable and novel electrochemical technique for the adjustment of various conductive substrates. Herein, BPE is performed to derive both of cathode and anode electrodes for the development of mediatorless/membraneless biofuel cell (BFC). On one hand, a preferable substrate for immobilization of bilirubin oxidase enzyme is prepared based on the electropolymerization of thiophene-3-carboxcylic acid (TCA) on an Au microfilm as a bipolar electrode. The resulted biocathode as novel bioelectrocatalyst offers a high electrocatalytic activity toward direct oxygen reduction reaction (ORR) with onset potential and current density of 0.55 V (vs. Ag/AgCl) and 867 μA cm^−2^, respectively. On the other hand, another analogous Au bipolar electrode is electroplated through BPE to derive Au nanostructures (AuNSs). This modified Au electrode is utilized as an anodic platform for immobilization of flavin adenine dinucleotide-dependent glucose dehydrogenase (FAD-GDH) enzyme aimed at electrocatalytic glucose oxidation. The prepared bioanode displays a current density of 2.7 mA cm^−2^ with onset potential of −0.03 V. Finally, the proposed bioanode and biocacthode in an assembled membraneless glucose/O_2_ BFC offers a power output of 146 μW cm^−2^ with open circuit voltage of 0.54 V. This novel BPE method provides disposable electrochemical platforms for design of novel sensors, biosensors or other devices.

## Introduction

Efficient electrical connection between redox sites of the biomolecules and electrode surface is a key factor in the development of bioelectrocatalytic applications such as enzymatic biofuel cells (EBFCs). An enzymatic BFC is an electrochemical system that utilizes enzyme substrates as catalysts for generation electricity from bioorganic fuel (typically glucose or other sugars) via bioelectrocatalytic reactions^1–3^. This bioelectrocatalytic process includes the oxidation of glucose at anode electrode by means of popular oxidoreductase enzymes such as glucose oxidases (GOx) or glucose dehydrogenases (GDH), and the reduction of oxygen at the cathode using multi-copper oxidase enzymes such as laccase or bilirubin oxidase (BOx).

High catalytic activity in direct reduction of O_2_, especially to H_2_O, in the neutral pH range and good stability, even at high temperatures^4^, makes BOx as widely interested cathodic bioelectrocatalyst^4–21^. Catalytic centers in the redox BOx enzyme are classified as type 1 (T_1_), type 2 (T_2_) and type 3 (T_3_). Compared to (T_2_,T_3_) Cu sites, T_1_ site is located at the outer layer of BOx in a hydrophilic substrate-binding pocket and responsible to electron transfer between electrode and T_2_,T_3_ sites^5–8^. Therefore, some of functional groups at the solid surfaces can effectively orientate the BOx enzyme molecules in a proper direction and lead to efficient electron transfer at biocathode^4–21^. The mechanism of direct electrone transfer (DET) between electrodes and multi-copper oxidases toward ORR is not fully understood^6,7,12^. Especially, it seems to be more complex than the mediated electron transfer^7^. However, the overall ORR catalyzed by BOx is a 4 electron transfer mechanism, since it is more efficient than two electron mechanism with H_2_O_2_ intermediate^6,7,12^.

On the other side, after the first discovering of FAD-GDH by Omura *et al*. in 2003, they reported the first glucose biosensor without requiring additional cofactors^22^. Lately, it has attracted more attention^23–30^, since unlike GOx, it is oxygen insensitive. As a consequence, O_2_ has a negligible influence on the catalytic process of FAD-GDH in a traditional BFC. Additionally, H_2_O_2_ which inhibits enzymatic reaction at cathode, is not produced during the anodic enzymatic reaction by FAD-GDH according to the following equations^3,29^:

1 $${\rm{FAD}} \mbox{-} {\rm{GDH}}+{\rm{d}} \mbox{-} {\rm{glucose}}\to {{\rm{FADH}}}_{{\rm{2}}} \mbox{-} {\rm{GDH}}+{\rm{d}} \mbox{-} {\rm{glucono}} \mbox{-} {\rm{1}},{\rm{5}} \mbox{-} {\rm{lactone}}$$

2 $${{\rm{FADH}}}_{{\rm{2}}} \mbox{-} {\rm{GDH}}\to {\rm{FAD}} \mbox{-} {{\rm{GDH2H}}}^{+}+{{\rm{2e}}}^{-}$$

Therefore, FAD-GDH can be utilized as bioanode without utilizing any separator in a BFC compartment.

The catalytic current produced by enzymatic redox reaction and resultant power density of BFCs is directly related to the electrical contact of enzymes with electrode surface^31^. Mainly, direct physical or covalent binding between enzyme molecules and electrode surface not display the proper electron transfer. So, for an effective interaction between enzyme and electrode, the electrode surface must be modified by the appropriate materials^32^. Conducting polymers (CPs), with excellent conjugated structures have been proposed as the promising substrate for fabrication and development of electrochemical sensors and biosensors^33,34^. Optimistic properties such as stability and high electrical conductivity of polymers can provide efficient electron transfer between electrode and enzymes, and sometimes CPs with extra functional groups have provided binding sites for the attachment of enzymes^34–37^.

Different carbon-based nanostructures such as carbon nanotubes^16,17,20,21,24,31,32,38^, graphene^18,28^, and carbon nanofibr^25^, metal and metal oxide, especially gold nanoparticles^2,9,13,23^, conducting polymers^34^, and so on, have been developed for supporting the enzymes toward bioelectrochemical applications. Most of those proposed procedures are costly and complicated. Moreover, in some cases, additional components called mediators are needed to facilitate electron flow between enzyme molecule and conducting material while, they are sometimes unstable and harmful in a bio-system application. Furthermore, the mediatorless electron transfer by enzyme can be more easily constructed in a membranless or miniaturized BFCs^39^. For a proper ET, the enzyme must be oriented in which way that its active sites get closer to the surface of electrode as short as possible^40^. The mediatorless electron transfer from GOx assisted by carbon nanotubes (CNTs) substrates is extensively reported^41,42^. But, it is well discussed in the recent bibliographies that direct oxidation of glucose by GDH can occur only in rare case. A few works related to direct glucose oxidation by FAD-GDH on gold nanoparticles and CNTs have been reported^23,31^. Hence, a rapid, simple and reliable method and materials to fabricate a proper substrate for easier ET by enzymes still is an important issue.

BPE has been recently explored as a versatile and reliable electrochemical technique for the constructing of conducting polymers or metal substrates^43–50^. The principles of bipolar electrochemistry is simple and composed of two driving electrode along with a bipolar electrode immersed in an electrolyte solution. The bipolar electrode is a wireless conductive object, which can simultaneously acts as anode and cathode electrodes^44^. In a cell containing a homogeneous electrolyte solution, when a sufficient driving voltage (ΔE_elec_) applied between two poles of the conductive object, the redox reactions take place simultaneously at its extremities wireless conductive BP object^43–50^. BPE provides particular advantages compared to conventional electrochemistry such as simple operation, which include a direct current (DC) power supply, low cost, no need to direct electrical connection and many electrodes can be controlled simultaneously with a single DC power supply^46,47^. BPE has been recently developed for electropolymerization of pyrrole^44^ and 3-methylthiophene^48,49^ or metal NSs^50^. Herein, BPE is proposed for adjusting the Au microfilm surface to fabricate a cathode and anode materials, because this technique has showed a great potential for the development of nanostructures. In order to provide the active site for effective loading of BOx for direct ORR, we selected thiophene with carboxylic acid polar functional group, thiophene-3-caboxylic acid (TCA), to produce a conduct polymer as transducers between enzyme and electrode surface. Also, with regard to effective immobilization of FAD-GDH on AuNSs in fabrication of bioanode^23^, another Au microfilm is electroplated through BPE to grow AuNSs based on dissolution/association at the anodic BP pols for direct oxidation of glucose. The fabricated bioelectrodes are assembled in membraneless biofuel cell system and produced significant power output.

## Experimental Section

### Materials and instruments

FAD-based GDH from Ex Aspergillus sp was purchased from SEKISUI, Japan. BOx from Myrothecium verrucaria (EC number 1.3.3.5), thiophen-3-carboxilic acid (TCA), Tetrabutylammonium hexafluorophosphate (TBAPF6, ≥99.0%), acetonitrile (ACN) (HPLC grade, ≥99.9%), p-Benzoquinone (≥98%) were obtained from Sigma Aldrich and Merck Co. The gold electrodes with high purity were purchased from commercial sources.

All electrochemical experiments were performed using a µAUTOLAB modular electrochemical system (ECO Chemie, Utrecht, The Netherlands), equipped with a GPES software in conjunction with a conventional three-electrode system. A gold (Au) electrode was employed as the working electrode and a platinum wire and Ag/AgCl/3 M KCl as the counter electrode and reference electrode, respectively. Scanning electron microscopy (SEM) images and energy dispersive X-ray spectroscopy (EDX) were obtained with a TESCAN MIRA3 HV operated at 20.0 kV. Z View software was used for fitting the impedance data.

### Overall Procedure for Bipolar Electropolymerization

The bipolar electrochemical system, which has been principally clarified in the literatures^42–49^, consist of two stainless steel driving electrodes (1 × 3 cm^2^ with 1 mm thickness) in a BP channel (1.2 × 3 cm) connected to the power supply (MASTECH DC Power Supply HY3005F-3) to provide the desired driving potential. The gold microfilm (1 × 2 cm^2^ with 0.1 mm thickness) as BP electrode was placed between the driving electrodes in a N_2_-saturated solvent containing electrolyte. The applied voltage was adjusted for a period of time in different potential to obtain the efficient condition. We prepared 2 BP electrodes, which TCA is grown on one of them for BOx immobilization and AuNPs on another electrode in purpose of FAD-GDH immobilization, according to the following procedures.

### Fabrication of Biocathode

The BP electrode and the driving electrodes were immersed in ACN solution containing 0.01 M of TCA, 0.006 M of TBAPF_6_ as supporting electrolyte, 0.005 M benzoquinone (BQ) as a sacrificial reagent and small amount of water (1–15% v/v). The BPE was done in several potentials (9–16 V) at different times, and based on the response of obtained biocathodes, the optimum condition was obtained. Also, for comparison of BPE with conventional cyclic voltammetry (CV), the electrodeposition of the TCA on the Au microfilm was carried out by CV method in the potential range of 0.0 V-1.6 V (versus Ag/AgCl) at a scan rate of 100 mV s^−1^. These modified electrodes were rinsed with ACN and ethanol solution to remove residues. The immobilization of BOx on the PTCA-modified Au electrode was performed by casting of 10 μL of 0.3 mg mL^−1^ dissolved BOx in 0.1 M phosphate buffer saline (PBS), pH 6, and was kept in refrigerator at 4 °C overnight.

### Fabrication of Bioanode

To generate AuNSs, an Au BP electrode was immersed into aqueous solution containing 0.002 M LiClO_4_ as supporting electrolyte in a designed BP channel. The BPE was carried out by applying the different voltage (10–30 V) to the stainless steel driving electrodes at different times (5–20 min). The prepared gold nanostructures were rinsed with deionized water several times. Then 10 μL of FAD-GDH solution dissolved in 0.1 M PBS, pH 7.1, was dropped onto the modified electrode, and was kept at 4 °C overnight.

The active surface area for both of biocathode and bioabode was calculated based on Randles-Sevcik equation:

3 $${{\rm{I}}}_{{\rm{p}}}=268600\,{{\rm{n}}}^{3/2}{{\rm{AD}}}^{1/2}{{\rm{Cv}}}^{1/2}$$

where, D represents the diffusion coefficient of K_4_Fe(CN)_6_, 7.6 × 10^−6^ cm^2^ s^−1^, C represents the concentration of K_4_Fe (CN)_6_, 5 × 10^−6^ mole cm^−3^, n is the number of electron transferred, equal to 1 and υ is the sweep rate of CV, 0.1 V s^−1^. By introducing specified amounts of each parameter, active surface area (A) was calculated ca. 0.08 cm^2^ for biocathode and 0.024 cm^2^ for bioanode.

## Result and Discussion

### Electropolymerization of thiophene-3-caroxylic acid through BPE

**Figure 1:**
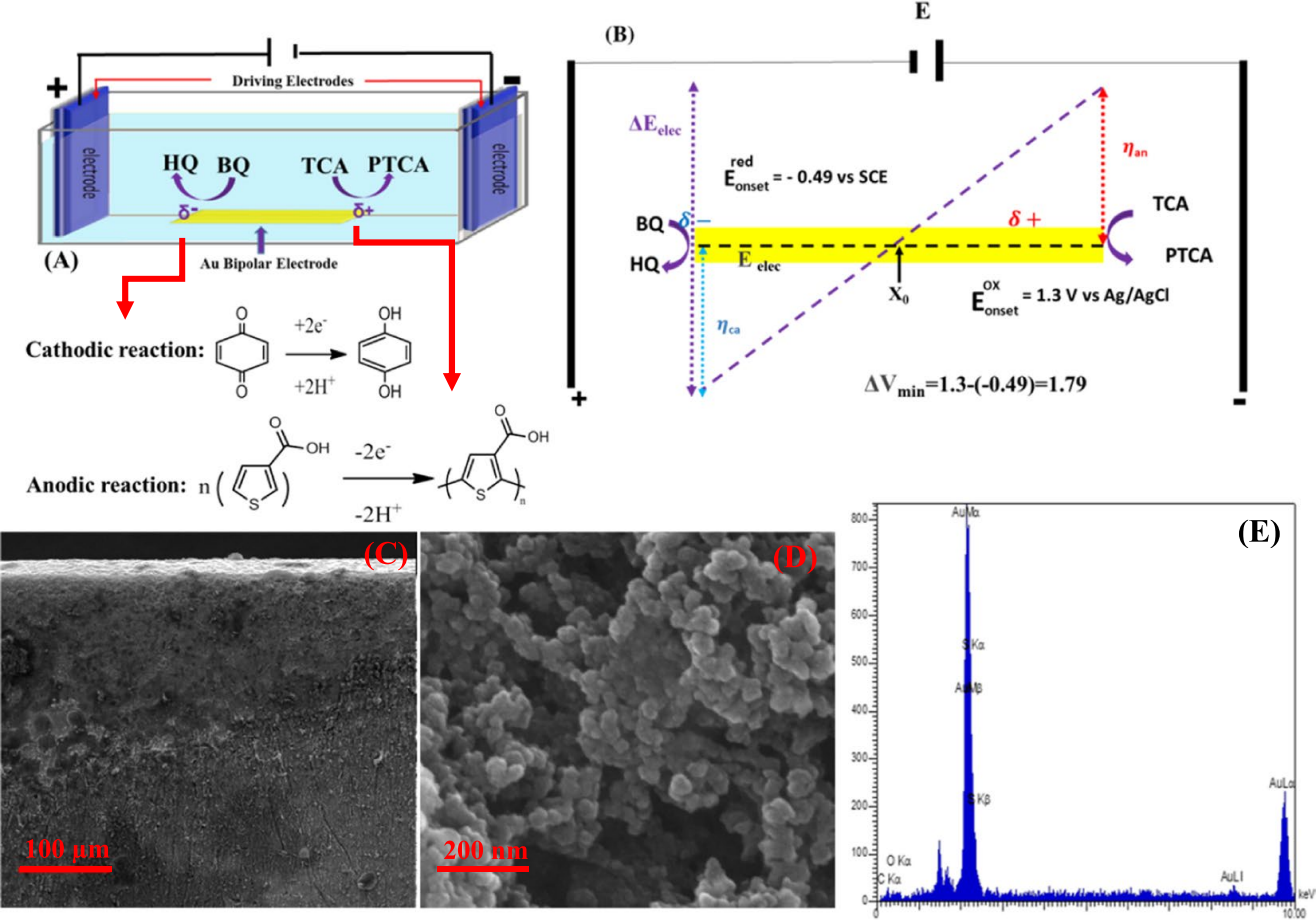
Schematic illustration of bipolar electrochemical setup for electropolymerization of TCA monomer (**A**) and distribution of anodic and cathodic overpotential (**B**). Different magnification SEM images related to PTCA synthesized through BPE (**C**,**D**). EDX depiction from anodic pole of Au electrode (**E**).

The BP electrochemical setup is schematically shown in Fig. 1A. The electropolymerization of TCA on the anodic pole of Au microfilm was taken place by applying potential to the driving electrodes, which were connected to the power supply. According to the BPE’s concept, electropolymerization of TCA monomer is initially taking place at the edge of anodic pole of BP electrode when a promising voltage is available, and then grows to reach the center of the electrode^43,44^. The first step of polymerization is the formation of TCA cation radicals, then the interaction between resulted cation radicals with anions of electrolyte is arisen. As a result, a dimer can be produced through losing two electrons and two protons. Since the oxidation of dimer occurs more easier than monomers^51,52^, polymer grows at the surface of Au BP electrode. Simultaneously, at cathodic pole of Au BP electrode, BQ is reduced to hydroquinone (HQ) when the acrossed potential over BP electrode is increased as high as difference in standard potential of the two involved redox couples (∆V_min_). Eventually, growth of polymer significantly depends on the magnitude of electric field, where the generated voltage between two stainless steel driving electrodes is calculated based on the following equation:

$${\boldsymbol{\Delta }}{{\bf{E}}}_{{\bf{elec}}}={{\bf{E}}}_{{\bf{tot}}}\cdot ({{\bf{L}}}_{{\bf{BPelec}}}/{{\bf{L}}}_{{\bf{channel}}}),$$

which can be controlled by strength of the external electric field (E_tot_), the length of the BP substrate (L_BPelec_) and the distance between the driving electrodes (L_channel_)^46,53–58^. According to the distance in potential peak position of cyclic voltammograms (CVs) related to the oxidation of TCA and BQ/HQ redox couples, with taking into account the potential drop of ∼10–15%, the minimum potential value, ∆V_min_, required to induce electropolymerization of TCA is about 1.79 V (Fig. 1B). To optimize the potential and time, E_tot_ was adjusted from 9 V to 16 V for 15 min and optimum potential was obtained to be 14 V based on the response of bioelectrode toward ORR. Similarly, time was adjusted at an applied constant potential of 14 V to the driving electrodes. It was found that a period of 20 min with applied potential of 14 V is sufficient to derive effective polymer/Au substrate (Fig. S1).

Figure 1C shows scanning electron microscopy (SEM) images of PTCA grown on the Au electrode surface. As can be seen, the high concentration of cloudy-like stuff, assigned the polymer, is observed at the edge of BP electrode, while it considerably decreased in the direction of middle part (the equilibrium potential) point of electrode. SEM image with higher magnification reveals the chain of particles with estimated size of 10–20 nm, which have been stacked together (Fig. 1D). The presence of carbon as well as sulfur atoms on the BP electrode surface is detected by EDX analysis (Fig. 1E), confirming the existence of the carbon-containing material on the Au surface.

**Figure 2:**
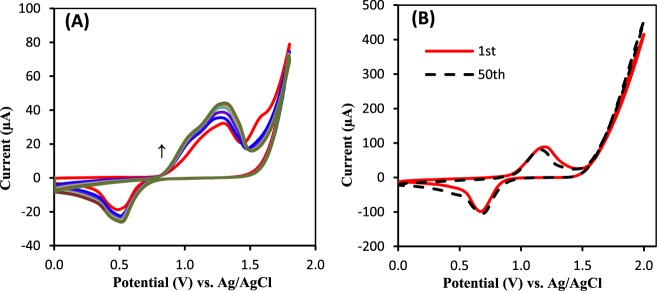
(**A**) Repetitive CVs attributed to PTCA polymerization on Au electrode in an ACN solution containing 0.01 M TCA + 0.1 M TBAPF_6_ + C_6_H_15_N with scan rate 100 mVs^−1^. (**B**) CVs of PTCA synthesized by bipolar electrochemistry in ACN + 0.1 M TBAPF_6_ (blank solution), scan rate 100 mV s^-1^, before and after 50 sequential cycles.

Because thiophene-3-caboxylic acid requires a high anodic potential window for carrying out the electropolymerization process, the electrochemical polymerization of corresponding monomer has mainly been performed in organic solvents due to possess higher potential window rather than aqueous solutions^57^. However, in some cases the property of synthesized polymers such as porosity and charge selectivity can be varied by small amount of water content^58^. Here, BPE was carried out in presence of various water content (1–15%). According to the catalytic response of our proposed BOx/BP electrode toward ORR, a 5% of water in ACN solution was selected as optimum value. In order to compare the BPE with conventional three electrode system, PTCA was also synthesized by CV and the electrocatalytic activity of resulting bioelectrode was compared to that of BP electrode. Figure 2A shows the CV behavior of 0.01 M TCA containing 0.1 M Bu_4_NH_4_PF_6_ in ACN solution at potentials range of 0.0 to 1.8 V. With increasing the number of CV scans the height of anodic peak at 1.25 V and corresponding cathodic peak at 0.55 V are simultaneously increased, while the unreversible anodic peak located at 1.65 V, assigned as oxidation of TCA monomer, is decreased due to mass loss of monomer at the surface of electrode, demonstrating the formation of TCA derivative such as PTCA. On the other side, when the prepared electrode through BPE was cycled in a monomer-free 0.1 M Bu_4_NH_4_PF_6_/ACN solution, a similar redox pair located at ~1.11 and 0.75 also can be seen, which they are more reversible than synthesized polymer by CV technique (Fig. 2B). These results indicate a similar reaction pathway for TCA through BPE or CV, but BPE lead to better cohesion of PTCA at the Au support and consequently, provide a proper substrate for enzyme immobilization. Those redox couple is stable even after 50 sequential cycles that it is a clear evidence to demonstrate the stability of generated polymer on solid surface of electrode.

**Figure 3:**
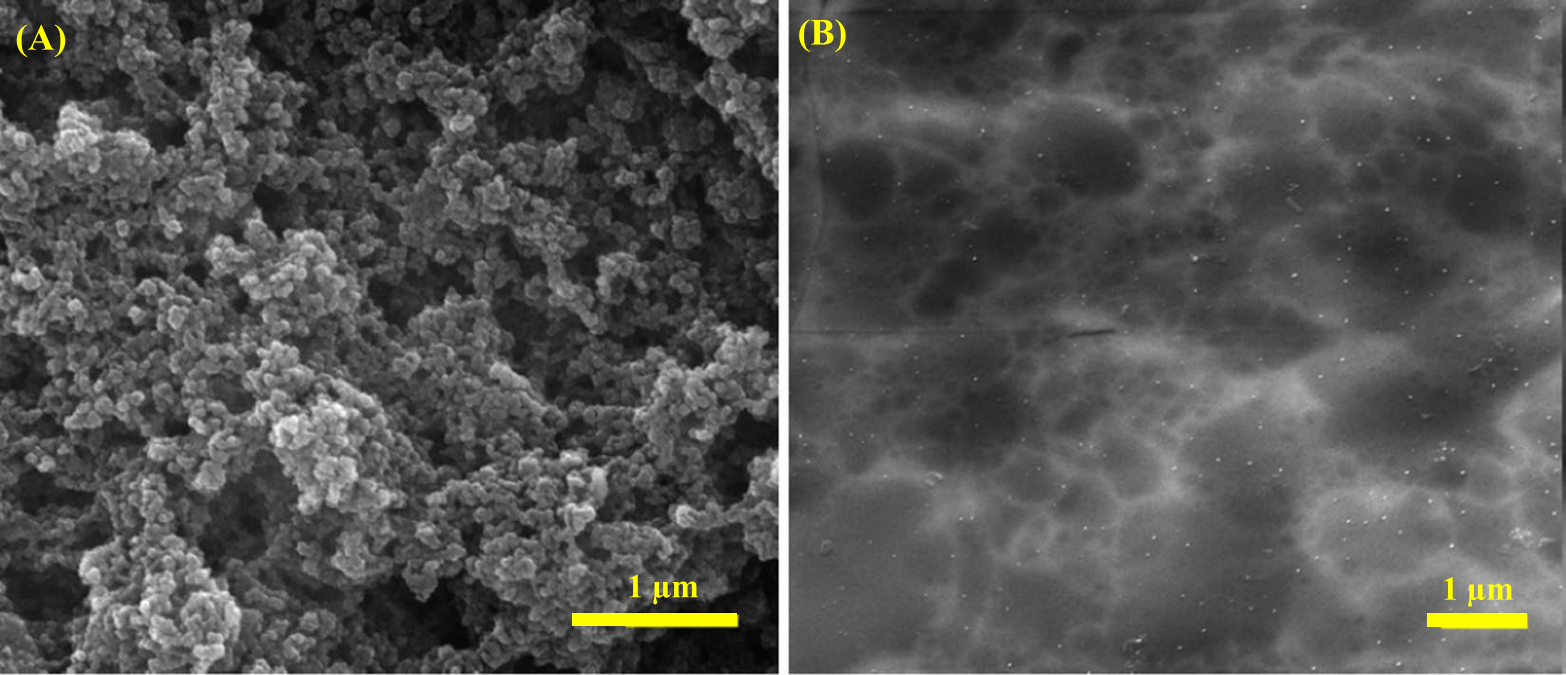
(**A**) SEM micrographs from PTCA-modified Au BP electrode before (**A**) and after BOx immobilization (**B**).

Enzyme attachment on the as-synthesized PTCA was done by casting of 10 μL of 0.3 mg mL^−1^ BOx in 0.1 M PBS, pH 6, and then kept in refrigerator at 4 °C overnight. It is expected that hydrophilic surface has been provided by carboxyl functional groups of polymer, and orients BOx in a proper way, which hydrophilic moiety of enzyme, contain T1 Cu center, closed to the electrode surface to offer DET^14–21^. Surface morphology of the electrode was evaluated before and after enzyme immobilization. Figure 3 shows SEM images of PTCA/BP electrode before (A) and after (B) BOx loading. It is observed that the surface morphology of PTCA/Au electrode has significantly changed after BOx immobilization, which attachment can be reinforced by the effective interaction between enzyme and functional groups at the electrode surface.

**Figure 4:**
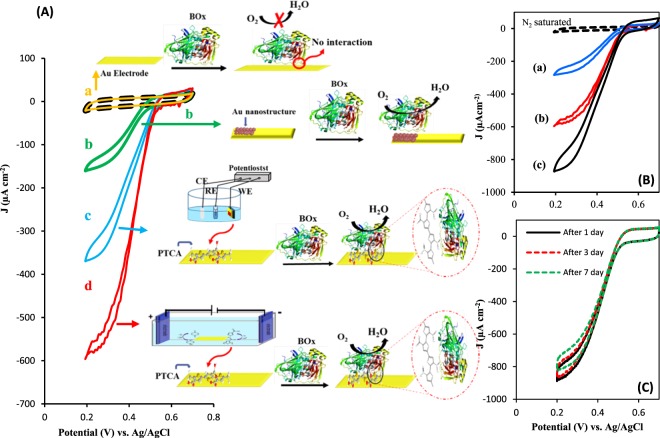
(**A**) Recorded CVs under N_2_-saturated (dash line) and O_2_-saturated attributed to BOx immobilized on bare Au electrode (a) AuNSs modified Au electrode (b) PTCA-Au electrode prepared by conventional three-electrode system (c) and bipolar electrochemistry method (d) along with schematic of their specific orientations. (**B**) Recorded CVs under N_2_-saturated (dash line) and O_2_-saturated for BOx immobilized onto PTCA/Au electrode prepared by BPE in ACN solution with different percent of water 0% (a) 5% (b) 15% (c). (**C**) CVs attributed to BOx immobilized on PTCA-modified Au electrode under O_2_-saturated after 1 cycle (solid line) and after passing some days (dash lines). Experimental condition: 0.1 M PBS, pH = 6, scan rate = 20 mV s^−1^.

**Figure 5:**
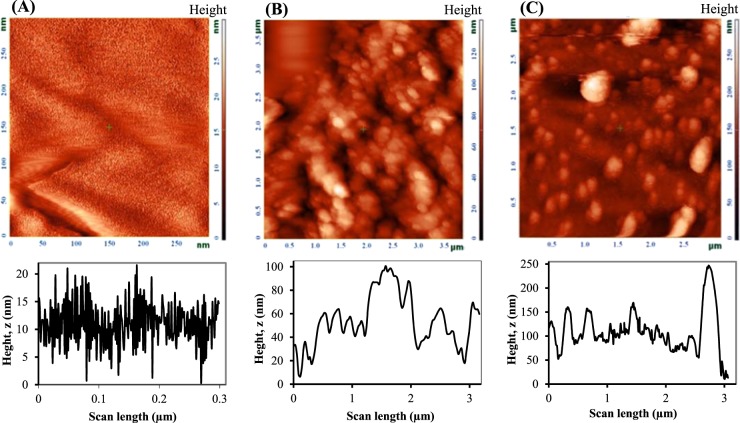
AFM-phase images (on top) along with roughness profiles (on bottom) of bare BP Au electrode (**A**), after BPE in an aqueous solution containing 0.002 M LiClO_4_ (**B**), after BPE in 95:5 acetonitrile/water containing 0.01 M of TCA, 0.006 M of TBAPF_6_ as supporting electrolyte, 0.005 M Benzoquinone (BQ) as a sacrificial reagent (**C**).

The behavior of electron transfer in each step of bioelectrode fabrication was investigated by recording CV and electrochemical impedance spectroscopy (EIS) in 0.1 M KCl solution, containing 1 mM [Fe(CN)_6_]^3−/4−^ redox probe (Fig. S2). Bare Au electrode exhibits a reversible redox peak related to [Fe(CN)_6_]^3−/4−^ (Fig. S2A, voltammogram “a”), while for the case of PTCA/Au BP electrode, the peak current is decreased because of increasing in electron transfer barrier against [Fe(CN)_6_]^3−/4−^ redox probe (voltammogram “b”). After enzyme immobilization on the PTCA/Au, the height of current intensity is decreased, while ΔE is increased, confirming BOx loading onto the electrode surface, that substantially hindering the electron transfer between electrode surface and [Fe(CN)_6_]^3−/4−^ redox couple (voltammogram “c”). The corresponding Nyquist plots from EIS at a polarization potential of 0.21 V vs. Ag/AgCl by varying the frequency of the voltage perturbation signal from 0.1 Hz to 100 kHz are also revealed the charge transfer resistance (R_ct_) of electrode in different step of fabrication (Fig. S2B). The obtained experimental data were fitted to Randle’s equivalent circuit (Fig. 5B, inset) and the results are given in Table S1, that includes the resistance of solution (R_s_), R_ct_, assigned as heterogeneous electron exchange of [Fe(CN)_6_]^3−/4−^ redox couple at the electrolyte, the Warburg element (W_o_), represent the semi-infinite diffusion of ions into the electrode in the lower frequency region, caused by surface inhomogeneity, roughness or electrode porosity, and constant phase element (CPE), representing the double-layer capacitance^59^. A minor amount of R_ct_ ca. 144 Ω for Au electrode is seen due to the high conductivity of Au metal electrode. After polymer deposition and BOx immobilization, R_ct_ is increased to 414 Ω and 960 Ω, respectively, due to hindrance of electron transfer from [Fe(CN)_6_]^3−/4−^ redox probe at electrode surface. Furthermore, during stepwise modification, W_o_ element is slightly increased from 987 Ω for bare Au to 1718 Ω for PTCA/Au, because of increasing in surface inhomogeneity, and then decreased when a homogenous layer of enzyme covers the surface of electrode. On the other hand, CPE value is significantly decreased from 270 to 55 µF s^α−1^ due to decrease in surface conductivity, while it is slightly increased after enzyme attachment because of contribution of enzyme in electron transfer at the surface of electrode. The resulted data measured by EIS and CV clearly approve stepwise construction of biocathode.

Electrocatalytic properties of the prepared bioelectrodes were examined by CV under oxygen saturated condition in 0.1 M PBS, pH 6, with scan rate of 20 mV s^−1^. Figure 4A represent the ORR activity of immobilized BOx on a bare Au electrode (a), an Au electrode prepared through BPE in the absence of TCA monomer (b), PTCA/Au electrode prepared by CV (c) and PTCA/Au electrode prepared by BPE (d). For bare Au (voltammogram a) no recognizable catalytic activity is observed, indicating BOx is not attached to the surface of the electrode. In the case of AuNSs, without PTCA, the surface roughness of Au electrode enables physical adsorption and entrapment of BOx and as a consequences, direct ORR can be seen (voltammogram b). Appending polar functional groups such as carboxylic acid to the aromatic compounds enhance the possible interactions such as Van Der-Waals and hydrogen bond or even covalent bonding between a solid surface and enzymes, lead to suitable orientation of immobilized enzyme for direct electron transfer^17–21^. However, the main drawback of those reports is the stepwise configuration of electrode to prepare a suitable substrate for attracting the enzyme. Hence, by this way the PTCA modified Au electrode through CV offers greater biocatalytic current density ca. 0.352 mA cm^−2^ at plateau region with decreased overpotential (550 mV vs. Ag/AgCl in pH 6 PBS) compared to that of BOx/AuNSs (voltammogram c). When PTCA electrogenerated through BPE, the current density of the proposed bioelectrode toward ORR reaches to 0.570 mA cm^−2^, shows 1.6 times enhancement in the catalytic activity than that of modified electrode by CV, which it may be due to the well-integration of polymer/Au BP electrode. The onset potential and current density related to this suggested bioelectrode toward ORR is comparable or better than that of previously reported (Table 1). In comparison, the present biocathode is an integrated polymer/Au substrate which prepared in a simple step with low cost of operation.

**Table 1:** Comparison of onset potentials and current densities of ORR from DET by different BOx based modified electrodes.

Electrode material	Onset potential (mV) vs. Ag/AgCl	Current density [µAcm^−2^]	Ref.
nanoporous gold electrodes	500 in pH = 7	800 µA cm ^**−2**^	^13^
MWCNT-modified gold electrode	485 in pH = 7	500 µA cm ^**−2**^	^14^
bilirubin/MWCNTs/GC	570 in pH = 6	270 µA cm ^**−2**^	^16^
PTCA/Au BP electrode	550 in pH = 6	867 µA cm ^−**2**^	This work

As mentioned above, small amount of water during electropolymerization of TCA enhances the catalytic activity of prepared bioelectrode. Figure 4B represents the catalytic current of different modified electrodes obtained in presence of different water content; 0% (a) 5% (b) 15% (c) during polymerization. A 5% of water displays catalytic current ca. 0.867 mA cm^−2^ with 1.5 folds enhancement than that of 0% water. Beside improvement in porosity of synthesized polymer in presence of water^58^, the impact of water on the surface morphology of the Au BP electrode (it will discussed in a section related to bioanode fabrication) in creating of AuNSs integrated with polymer, may be another reason in enhancement of catalytic activity of resulted bioelectrode. However, the larger amounts of water content can inhibit the proper polymerization, where by increasing the water content to 15%, the catalytic current is drastically decreased.

The stability of the modified electrode is one of the significant parameter in utilization over time. The modified electrode at the first time displayed 0.975 mA cm^−2^, but after several CV scan, it has lost about ~13% of catalytic current, which it can be because of detachment of weakly adsorbed enzyme molecules. Therefore, before of biocathode application in a BFC, it was stabilized through several CV cycles. Compared to the first stabilized BOx-modified electrode, with the passage of 7 days the modified electrode has lost just about 5% of catalytic response (Fig. 4C). Such stability is almost comparable to the previously reported works^13–21^. However, the electrocatalytic response can be more stable for a longer time by modifying the biocathode with some stabilizers such as P017-epoxy^13^.

### Preparation of AuNSs through BPE as electrochemical platform for FAD-GDH immobilization

In the next set of experiments, the oxygen-insensitive flavoenzyme, FAD-GDH, was employed to design an anodic bioelectrocatalyst. It was immobilized on the AuNSs substrate, prepared through BPE according to a principle as described above. Hence, BPE containing an Au BP electrode was located between two driving electrodes in an aqueous solution containing 1 mM LiClO_4_ electrolyte. In the previous section, prior of Au, the oxidation of TCA was occurred. Here, in the absence of TCA monomer in aqueous solution, AuNSs are electrogenerated through BPE on the anodic pole of Au microfilm, when an optimum potential (20 V) at optimum time (20 min) is applied to the driving electrodes. The activity of the enzyme is associated to the effective adsorption of enzyme onto electrode surface. Here, the electroplated Au microfilm through BPE could leads to the surface roughness incorporated by AuNPs, which effectively attracts enzyme for proper mediatorless ET^15^. AFM topological analysis as an effective way was applied to reveal the surface roughness of PB electrode before and after BPE. For a bare BP Au electrode, the AFM-phase image and height profile reveals the height of peak-to-valley ca. 21 nm (Fig. 5A). Through BPE in an aqueous solution containing 0.002 M LiClO_4_ (Fig. 5B) or after BPE in 95:5 ACN/water containing 0.01 M of TCA, 0.006 M of TBAPF_6_, 0.005 M BQ (Fig. 5C), the height of peak-to- valley is increase to 100 nm and 245 nm, respectively. Commonly, surface roughness is estimated by measuring the root mean square (RMS), which can be calculated according to the following equation^60^:

4 $$RMS={\sum }_{i=1}^{N}{[\frac{({z}_{i}-\bar{z})}{N}]}^{1/2}$$

where $\bar{z}$ is the average height of the surface profile is defined as:

5 $$\bar{z}={\sum }_{i=1}^{N}{z}_{i}$$

z_i_ represents the surface height at each data point on the surface profile and N is the number of data points. Accordingly, RMS is obtained to be 7.0, 12.9 and 24.2 for bare Au, AuNSs and PTCA/Au, respectively, showing ca. 2 and 3 folds increasing after BP operation in the absence and presence of TCA, respectively.

**Figure 6:**
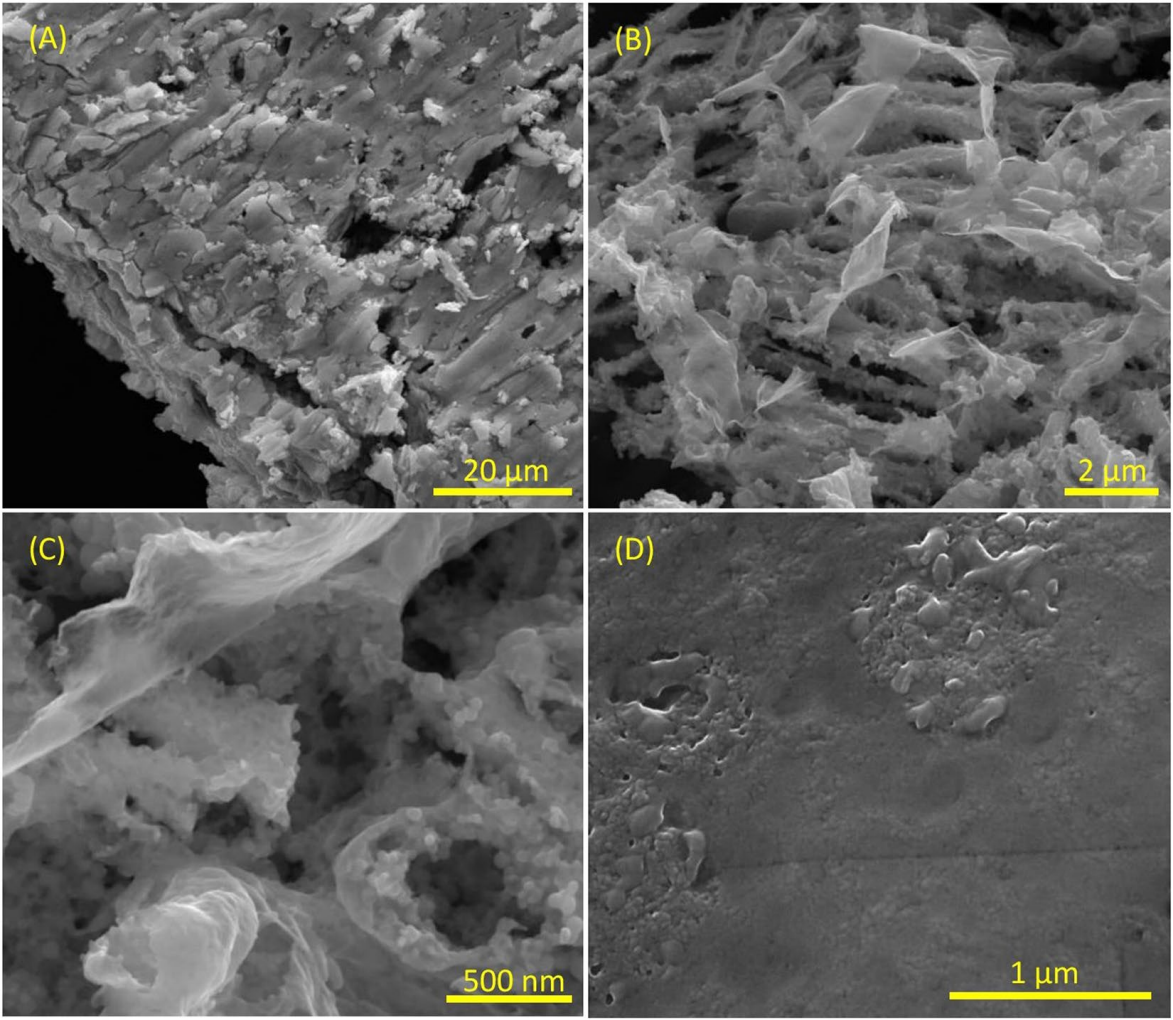
SEM images of the electrogenerated AuNSs resulted from BPE on Au microfilm with different magnifications (**A**–**C**) and after FAD-GDH immobilization (**D**).

Large scope SEM micrograph from the anodic pole of Au substrate demonstrates various micro-cracking in different directions (Fig. 6A). SEM images with higher magnification reveal various irregular flaks on top of brambles, resulted from corrosion of Au surface through BPE (B,C). After enzyme loading, almost a smooth surface, demonstrated the enzyme, is seen (D). Additional SEM images before and after enzyme loading with different magnification can be seen in Fig. S3.

Alike the biocathode, fabrication of bioanode was also studied by recording CVs and EIS to follow the changes in electron transfer barrier related to [Fe(CN)_6_]^3−/4−^ probe. A couple of well-defined reversible peak could be observed by AuNSs modified-Au electrode, which after enzyme attachment the peak current is evidently decreased and ∆E_P_ is increased to 0.22 V (Fig. S4A). Fig. S4B shows impedance spectra recorded by AuNSs/Au PB electrode (a) and after FAD-GDH immobilization (b). The impedance spectra were fitted to a modified randle’s equivalent circuit as shown in the inset of Fig. S4B, and the obtained data are shown in Table S2. Compared to the bare Au, the value of R_ct_ for AuNSs, related to the interfacial electron transfer resistance, is increased due to the formation of metal oxides NSs. After enzyme loading, R_ct_ is significantly increased ca. 7 times, indicating that the enzyme molecules act as a barrier against electron transfer of Fe(CN)_6_^3−/4−^ redox to the electrode surface. These observations along with change in W_o_ and CPE confirm the change in the interfacial resistance and diffusion layer of electrode-solution contact during electrode fabrication in the ascribed processes.

**Figure 7:**
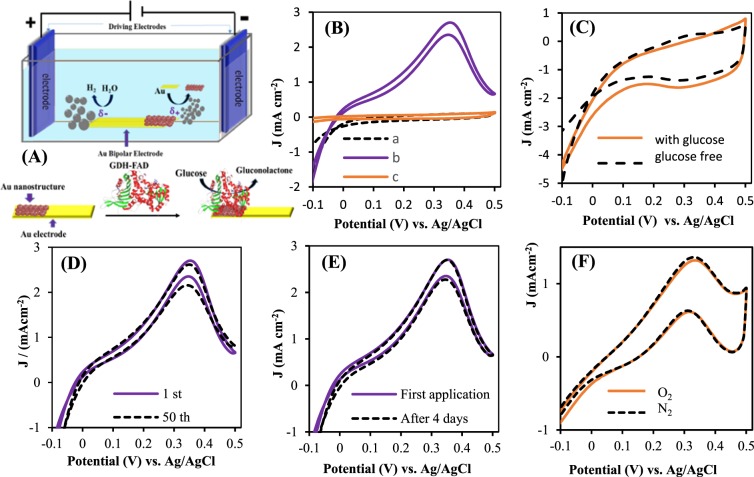
(**A**) Schematic design of bipolar electrochemical system for generation of AuNSs and immobilization of FAD-GDH enzyme on it. (**B**) Recorded CVs with FAD-GDH/AuNSs BP electrode in absence (dash line,a) and 0.1 M glucose (solid line, b) GDH-FAD on bare Au in presence of glucose (dark orange,c). (**C**) CVs of AuNSs in absence (dash line) and presence of 0.1 M glucose (solid line). (**D**) Recorded CVs by FAD-GDH/AuNSs in 0.1 M glucose after 1^th^ (solid line), 50^th^ and 10^th^ (dash lines). (**E**) CVs of first application (solid line) and after 4 days (dash line) of bioelectrode. (**F**) FAD-GDH/AuNSs in presence of 0.05 M glucose in N_2_ saturated and O_2_ saturated solution. Experimental condition: 0.1 M PBS, pH = 7.1, scan rate 10 mV s^−1^.

The bioelectrocatalytic properties of resulted bioanode by suggested BPE (Fig. 7A) were examined by recording CVs in 0.1 M PBS solution, pH 7.1, with scan rate of 10 mV s^−1^ at a potential range of −0.1 to 0.5 in the absence and presence of glucose. The differences in resulted CVs could be assigned as electrocatalytic response of bioelectrode. The prepared FAD-GDH/AuNSs bioelectrode catalysis the oxidation of glucose with onset potential of −0.03 V and displays an electrocatalytic anodic current ca. 2.7 mA.cm^−2^ in the presence of 0.1 M glucose (Fig. 6B, curve “b”). Whereas, for immobilized enzyme on bare Au electrode (Fig. 7B, curve “c”) and AuNSs without enzyme (Fig. 7C), no obvious electrocatalytic oxidation of glucose are observed, representing the role of enzyme and AuNSs in bioelectrocatalytic oxidation of glucose. These results point to the fact that BPE as a powerful technique leads to the proper substrate, which strongly entrap the GDH enzyme. The onset potential and current density related to suggested bioelectrode toward glucose oxidation is almost comparable to the previously reported GDH-based modified electrodes (Table 2).

**Table 2:** Comparison of onset potentials and current densities related to glucose oxidation at different GDH based modified electrodes.

Electrode material	Onset potential (mV) vs. Ag/AgCl	Current density (mA cm^−2^)	Ref.
NAD-GDH/NB/Den/MWCNTs NH_2_/GC	−225 in pH = 7	0.240	^17^
FAD-GDH/menadion/CNT	−200 in pH = 7	1.66	^24^
FAD-GDH/rGO/PTZ-O/GCE	−250 in pH = 6.5	0.5	^28^
FAD-GDH/SWCNT	+100 in pH = 7.4	1.2 at +0.6 V	^31^
FAD-GDH/AuNSs	−30 in pH = 7.1	2.7	This work

Stability of the FAD-GDH modified electrode was studied by comparing of first CV scan of 0.1 M glucose with 50^th^ and 100^th^ CV scan (Fig. 7D) and also with recorded CV scan of that electrode stored in refrigerator at −4 °C for 4 days (Fig. 7E). A rather steady state catalytic response is found that represent the storage stability of modified electrode. Additionally, it is well-known that bioelectrocatalytic activity of FAD-GDH enzyme for glucose oxidation is insensitive to oxygen, which means no interference from oxygen on the performance of bioanode. Figure 7F show the comparable CV curves attributed to glucose oxidation under O_2_ or N_2_ atmosphere, illustrating that GDH/Au NSs bioanode could be employed in membraneless glucose/O_2_ biofuel cell.

As mentioned above, the mechanism of mediatorless is not fully understood and it is more complex than that of mediator-based electron transfer^6,7,12^. DET from FAD-GDH on AuNSs has been seen by O. Yehezkeli *et al*.^23^ and they recorded the same CVs related to glucose oxidation like that we obtained in this work. But they have not provided any discussion about the mechanism of ET. Generally, the catalytic behavior of FAD-GDH redox center can be alike that mechanism we referred in introduction section^3,29^. Moreover, the possible enzymatic electron transfer mechanisms from redox center to electrode surface have been reviewed by many researchers^61,62^, which electron tunneling (or superexchange) and electron hopping mechanisms for DET have been highlighted. On one hand, through favorable orientation of enzyme at electrode surface, which the redox center of enzyme is in a lowest distance to the electrode surface, electron tunneling going forward. Accordingly, electrons are transported through cross bridge formation (assisted by different functional groups of enzyme) between redox active site of enzyme and materials at electrode surface, when an overlap occurs between donor and acceptor orbitals (enzyme and electrode). Such bridge orbitals of functional groups facilitate electronic communication between primary donor and final acceptor and lead to electron tunneling. On the other hand, when the distances increased up to the upper limit for electron tunneling, the sequential ET (hopping) is the decisive process. According to this mechanism, a series of short ET steps concludes the overall ET between primary electron donor and final electron acceptor. However, the overall electron transfer can be limited to the first monolayer of enzyme molecules which attached to the electrode surface.

**Figure 8:**
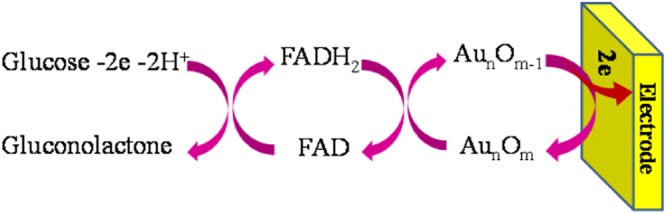
The proposed direct electron transfer of FAD-GDH on Au nanostructures.

With these explanations, the ET mechanism in multi-center enzymes such as BOx is so complicated, and in the present work, electrons may be transferred through both of electron tunneling and hopping processes. On the other side, based on our understanding, the penetration of FAD-GDH into irregular flaks/bramble-like AuNSs is a key factor for tightly interaction between enzyme molecules and electrode surface in 3D direction via terminated sulfur functional groups of enzyme, which they have a respectable affinity to Au species. As a consequence, the efficiency of enzyme immobilization is increased and electrons can be transferred through tunneling mechanism. But, the voltammogram shape in higher overpotential (~0.3 V, Fig. 7) is look like the voltammeric behavior of non-enzymatic glucose oxidation at noble metal nanostructures in acidic or basic solutions. The mechanism of non-enzymatic glucose oxidation at some of noble metal nanostructures has been described somewhere^63–65^. For example, a small amount of iron beside Pt could act as an mediator between glucose and Pt electrode^63^. In the case of Au, it has been proposed that the voltammogram peak in the backward scan can be attributed to the re-oxidation of reduced Au_n_O_m_^64,65^. Therefore, it can be understood that AuNSs as an inorganic redox probe is also contribute in DET of FAD-GDH according to the Fig. 8.

To conclude these sections, this BPE method could be recommended, while it needs a simple and low cost operation procedure and rapidly offers the desired modified surfaces by organic polymer films such as PTCA or metal nanostructure such as AuNSs. BOx enzyme can be attached to the polar functional groups of resulted PTCA from the appropriate side. On the other side, FAD-GDH molecules penetrate into irregular flaks/bramble-like AuNSs. As a consequence, such prepared modified surfaces intensely hold enzymes and offer direct electron transfer in O_2_ reduction and glucose oxidation, respectively. We have compared the rate of electron transfer at the electrode surface (K_et_) for cathode and anode according to the following equation^66^:

6 $${{\rm{J}}}_{{\rm{0}}}={{\rm{nFk}}}_{{\rm{et}}}{\rm{C}}$$

where C is the concentration (O_2_) = 1.25 × 10^−6^ (mole cm^−3^), C (glucose) = 100 (mole cm^−3^), n = 4 for O_2_ reduction and 2 for glucose oxidation, F = 96500 C mole^−1^ and J_0_ is the exchange current from Tafel intercept of polarization curves for O_2_ reduction and glucose oxidation. Accordingly, k_et_ for cathode and anode were obtained ca. 3.85 × 10^−6^ cm s^−1^ and 1.32 × 10^−4^ cm s^−1^, respectively. Lower K_et_ of cathode than the anode can be attributed to the differences in surface/interface interaction of enzymes with electrodes, and also lower conductivity of PTCA rather than the AuNSs, as seen by EIS.

### Glucose/O_2_ BFC establishment

Prior of BFC establishment, the effect of pH and different concentration of O_2_ and glucose were examined to choose the optimum conditions. Upon successive increasing of oxygen concentration the current density is increased (Fig. S5A). However, due to the successive application of electrode or side reduction products such as O_2_ to H_2_O_2_, the plateau limit not reaches as high as uninterrupted O_2_ saturation.

Figure S5B depicts the typical CVs of bioanode in the presence of variable concentrations of glucose. As shown, the catalytic peak current is increased as a function of increasing glucose concentration until it reaches to 100 mM. Here, BFC is studied at continues O_2_ saturated in presence of 0.1 M of glucose and also physiological-like glucose concentration.

**Figure 9:**
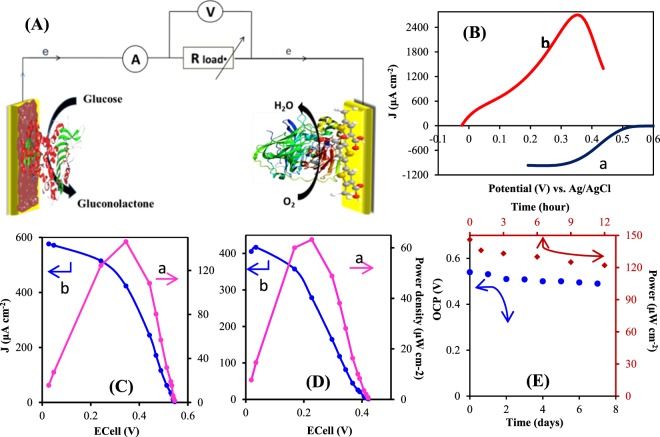
(**A**) Schematic of BFC compartment using FAD-GDH/AuNSs/BP bioanode and BOx/PTCA/Au BP biocathode. (**B**) Polarization curves for BOx/PTCA/Au PB electrode in oxygen saturated solution with scan rate of 20 mVs^−1^ (a) and FAD-GDH/Au NSs/Au BP electrode bioanode in 100 mM glucose at scan rate10 mV s^−1^. (C) Power density (a) and polarization curve (b) of glucose/O_2_ membranless BFC in 0.1 M PBS, pH 7.1 containing 0.1 M glucose saturated with O_2_. (D) Power density (a) and polarization curve (b) of glucose/O_2_ membranless BFC in human-like glucose condition (5 mM). (**E**) Theoretical OCV stability of BFC based on the distance in onset potentials of anode and cathode (circle points) and experimental study (in 100 mm of glucose and O_2_ atmosphere) of power output stability (square points).

To investigate the performance of prepared bioelectrodes in various pHs, PTCA-BOx and FAD-GDH/AuNSs modified electrodes are employed in different pHs near to the physiological condition. As can be seen in Fig. S5C, due to participation of proton in ORR, catalytic current is slightly increased with decreasing pH. In contrast the activity of FAD-GDH bioelectrode in pH = 7 is larger than pH = 6, showing more activity of this enzyme in physiological-like condition (Fig. S5D). The onset potential of both reactions toward oxygen reduction and glucose oxidation by increasing pH, is shifted to negative values, signifying that H^+^ is involved in the both reactions. In view of these results, the nearest pH to physiological condition was selected and BFC is established in pH = 7 at room temperature, which is schematically shown in Fig. 9A.

According to the onset potentials of glucose oxidation (−0.03 V) and O_2_ reduction (0.53 V) at pH = 7 (Fig. 9B), a theoretical open circuit voltage (OCV) as high as 0.56 V is expected. Figure 9C displays the current-voltage polarization curve along with power-voltage behavior resulted from 0.1 M glucose/O_2_ BFC, achieved by varying the external resistances. This BFC offers a maximum power density ca. 146 μW cm^−2^ at 345 mV and current density of 576 µA cm^−2^ with the OCV (0.54 V) as much as theoretical value.

Owing to the future of BFC applications in physiological fluids, the performance of this suggested BFC was operated under 5 mM glucose as human physiological concentration. In this condition, the maximum power density and maximum current density in the BFC is 63 μW cm^−2^ and 405 μA cm^−2^, respectively (Fig. 9D). Interestingly, this power output is two times higher than the recently reported FAD-GDH/BOx BFC, where Au nanoparticle and CNT have been used as substrate for immobilization of GDH and BOx, respectively (32 μW cm^−2^)^23^. On the other hand, the obtained value is slightly lower than that power output reported by our group worker (108 μW cm^−2^) which safranin has been used to mediate the electron transfer between NAD-based GDH and MWCNTs/Denderimer/GCE^16^, and lower than that value reported by Ji *et al*.^67^ (124 μW cm^−2^), which they proposed a membrane-based BFC using Fe_3_O_4_/carbon nanofiber/gold nanoparticle. But, the mediatorless electron transfer in a membraneless BFC is in higher significant rather than mediator-based systems due to the side effect and lower stability of chemical mediators and complicated establishment of membrane-based BFC. Nevertheless, this membranless/mediatorless system in high concentration level of glucose offers more power output than the recently reported membranless/mediatorless BFC, which in that DET from GOx has been obtained (102 µW cm^−2^).

The response stability of the BFC was investigated in different times. The theoretical OCV based on the distance in onset potentials of anode and cathode is presented in Fig. 9E (circle points). With passing of 7 days, a slight decreasing in OCV is seen. Additionally, experimental study of power output (in 100 mM glucose and O_2_ atmosphere) during 12 hours shows a little decreasing (ca. 16%) in power output (Fig. 9E, square points), that it may be because of the side effects of bioanode on biocathode and vice versa. However, the rapid and simple operation of this proposed BPE in preparing the effective substrates compensates the imaginable drawbacks. This mediatorless and membraneless system can be a good candidate in the flow-through BFC systems reported by many workgroups^68,69^, since the proposed substrates consist of modifiers that strongly incorporated with electrode. Subsequently, there is no possibility of modifier detachment from electrode or decreasing of mediator concentration in a flow-through system.

## Conclusion

In this study bipolar electrochemistry as a simple and efficient method was proposed for electropolymerization of thiophen-3-carboxylic acid as well as electrogeneration of AuNSs grown on the bipolar Au microfilms. The obtained polymer with carboxyl functional groups at the solid state electrode provided powerful interaction with bilirubin oxidase enzyme to apply in electrochemical oxygen reduction reaction. Compared to the PTCA, that prepared with conventional methods, the onset potential and current density was significantly improved. On the other side, a bioanode was prepared by immobilization of FAD-GDH on the resulted AuNSs/Au BP microfilm and electrocatalytic oxidation of glucose was achieved. The electron transfer by BOx and FAD-GDH without performing any chemical mediator is the great importance of this work. A BFC compartment resulted from suggested biocathode and bioanode have offered a voltage of 540 mV with power density of 146 μW cm^−2^. The results of this study clearly indicate that BPE can be a pragmaticand powerful synthesis method in organic and inorganic electrochemistry to drive various micro or nanostructures designed for extensive catalytic performances, biosensing tools and related devices.

## Electronic supplementary material

Supplementary Information
