# Supplementary material for: Direct Enzymatic Glucose/O2 Biofuel Cell based on Poly-Thiophene Carboxylic Acid alongside Gold Nanostructures Substrates Derived through Bipolar Electrochemistry
Source: Sci Rep. 2018 Oct 10;8:15103. doi: 10.1038/s41598-018-32893-2 (PMC6180125; doi:10.1038/s41598-018-32893-2)
Supplement: Supplementary file 1 — Supplementary Information [file 41598_2018_32893_MOESM1_ESM.docx]

**Supporting information**

**Direct Enzymatic Glucose/O_2_ Biofuel Cell based on Poly-Thiophene Carboxylic Acid alongside Gold Nanostructures substrates Derived through Bipolar Electrochemistry**

Fereshte Gholami,^‡^ Aso Navaee,^‡^ Abdollah Salimi,^‡†*^ Rezgar Ahmadi^‡^ , Azam Korani^‡^, Rahman Hallaj

^‡^*Department of chemistry, university of Kurdistan, 66177-15175, Sanandaj- Iran.*

^‡^*Research Centre for nanotechnology, University of Kurdistan, 66177-15175, Sanandaj- Iran,*

*Corresponding Author:*

*Tel: +98-87-33624001, Fax: +98-87-33624008; e-mail:absalimi@uok.ac.ir,* [*absalimi@yahoo.com*](mailto:absalimi@yahoo.com)


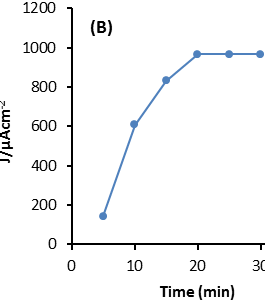

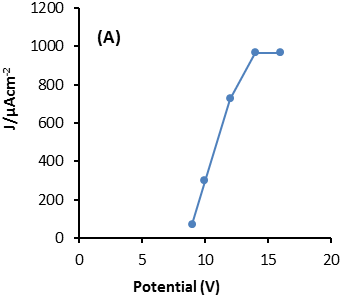


**Figure S1:** Effect of potential (A) and time (B) of BPE operation on the response of prepared BOx/PTCA/Au electrode toward ORR.

**Figure S2:** CVs (A) and EIS (B) graphs of Au (a) Au/Polymer (b) Au/Polymer/BOx (c) in KCl 0.1 M containing 5 mM of [Fe(CN_6_)]^3-/4-^

**Table S1:** Impedance fitting parameters for BP electrode at different steps of biocathode fabrication.

| Element | Au | PTCA/Au | BOx/PTCA/Au BP |
| --- | --- | --- | --- |
| R_ct_ (Ω) | 150 | 414 | 960 |
| W_o_-R (Ω) | 978 | 1718 | 1121 |
| CPE-T (µF s^α-1^) | 270 | 55 | 73 |


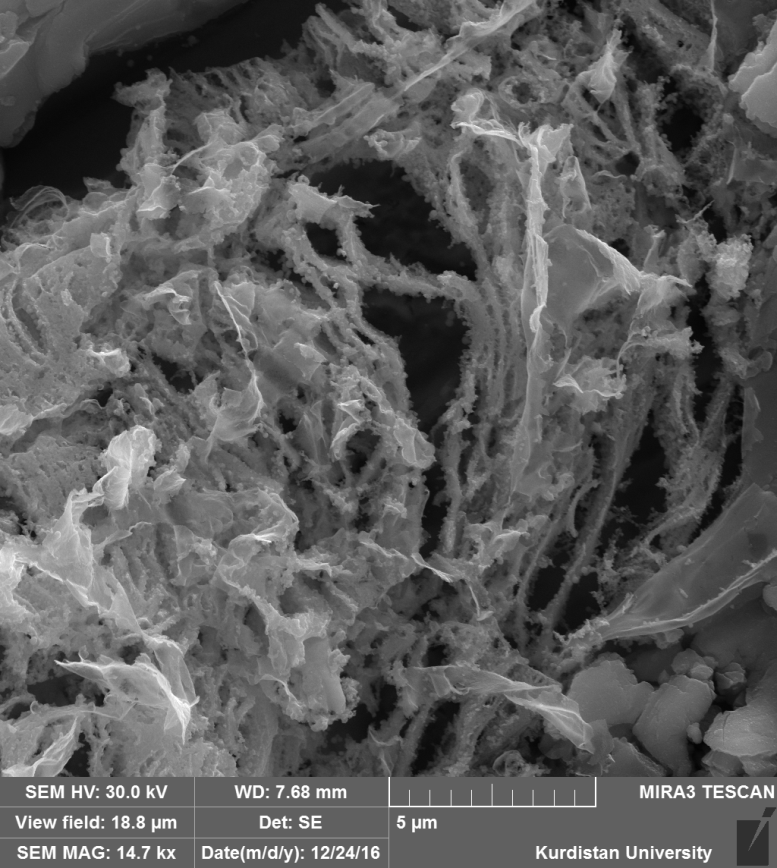

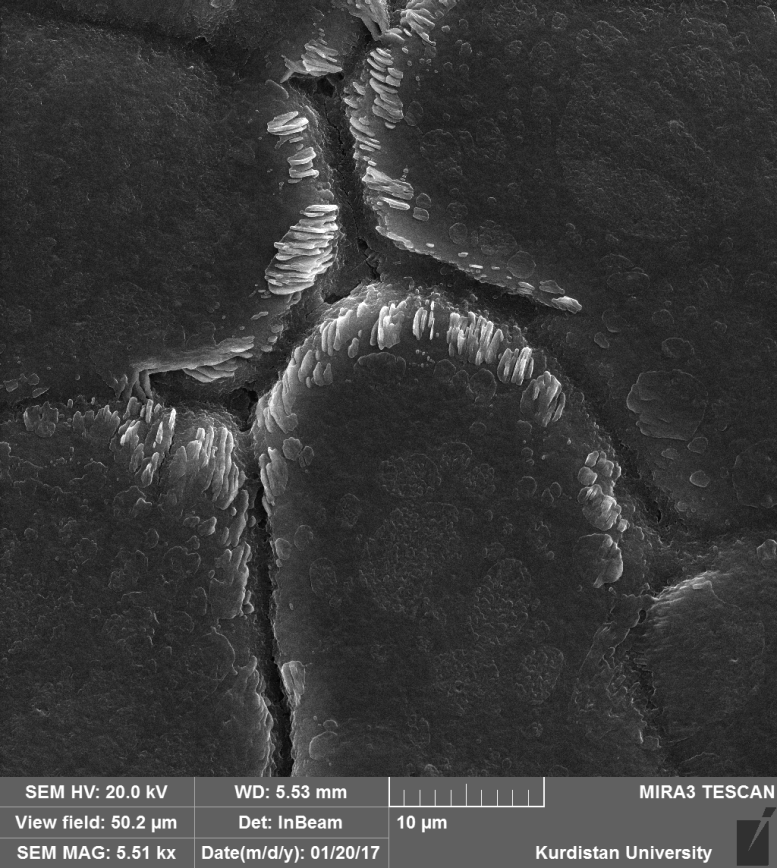


**Figure S3:** SEM micrograph of Au nanostructures resulted under BPE (left hand) and after enzyme immobilization (right hand).


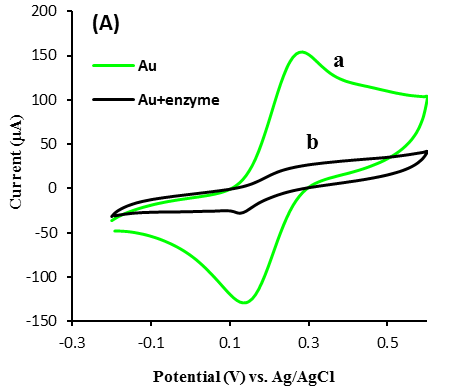

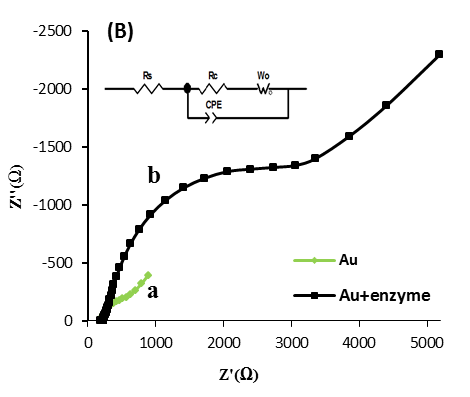


**Figure S4:** CVs (A) and EIS (B) graphs of Au NSs modified Au electrode (a) FAD-GDH/Au NSs modified Au electrode (b) in KCl 0.1 M containing 5 mM of [Fe(CN_6_)]^3-/4-^.

**Table S2:** Impedance fitting parameters for BP electrode at different steps of bioanode fabrication.

| Element | Au | Au nanostructures | FAD-GDH/Au nanostructures |
| --- | --- | --- | --- |
| R_ct_ (Ω) | 139 | 574 | 3652 |
| W_o_-R (Ω) | 978 | 315 | 768 |
| CPE-T (µFs ^α-1^) | 254 | 327 | 39 |

**Figure S5:** Recorded CVs, attributed to (A) BOx/PTCA/Au PB electrode in pH=6, scan rate 20 mV s^-1^ for different bubbling times of oxygen (5, 10, 15, 20, 25, 30, 35, 40, 45, 50, 55, 60, 65, 70 s, (B) FAD-GDH/Au nanostructure in PBS pH=7.1, scan rate 10 mVs^-1^) in absence of glucose (dash line) and presence of 25 (a), 50 (b), 100 (c) mM of glucose, (C) BOX/PTCA/Au BP electrode under N_2_-saturated (dash line) and O_2_-saturated (solid line) in 0.1 M PBS pH=5 red curve, pH=6 dark curve and pH=7 pink curve with scan rate 20 mVs^-1^, and (D) represents FAD-GDH/Au NSs modified Au electrode in 0.1 M PBS with scan rate 10 mVs^-1^ in presence of 0.05 M glucose in pH=7.1 dark curve and pH=6 red curve.
